# Unusual Pauses In Sinus rhythm And Intermittent 2 to 1 AV Block In A Patient With Concealed His Extra Systoles - A Rare Cause Of Bradycardia

**Published:** 2010-03-05

**Authors:** Siva K Mulpuru, Ravi Diwan, Dayana Eslava Manchego, Cesare Saponieri, Balendu Vasavada

**Affiliations:** 1 Fellow, Cardiovascular Diseases, Beth Israel Medical Center and Long Island College Hospital, Brooklyn, NY; 2 Attending, Cardiac Electrophysiology, Division of Cardiology, Long Island College Hospital, Brooklyn, NY

**Keywords:** His extra systoles, functional AV block

## Abstract

A 56 year old male with a past medical history of hypertension and dyslipidemia presented with recurrent dizziness. Routine EKG was performed, which suggested frequent junctional extra systoles with compensatory pauses. During telemetry periods of 2:1 block with effective ventricular rate of 34 bpm was observed. His bundle study suggested frequent His extra systoles causing functional AV block. Treatment with anti-arrhythmic medication, paradoxically improved AV block and symptoms in our patient.

## Case Report

**Figure 1:**
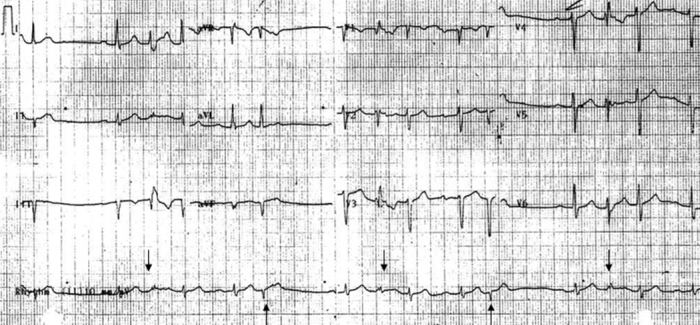
EKG on presentation shows normal sinus rhythm with frequent extra systoles. Extra systoles after a compensatory pause exhibit RBBB morphology suggestive of aberrancy (downward arrows). Narrow complex extra systoles (upward arrows) were thought to be junctional in origin.

**Figure 2:**
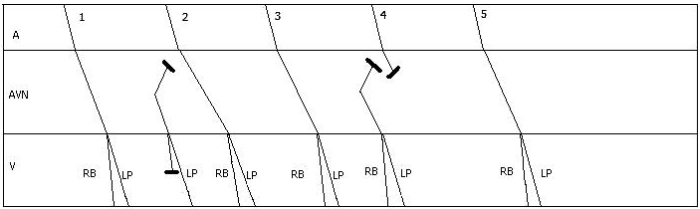
Ladder diagram to illustrate beats between pauses. The first beat is conducted with first degree AV block and LAFB. His extra systole is conducted with RBBB (Ashman aberrancy). Prolongation of PR interval of the next sinus beat can be explained by concealed conduction from His extra systole. 3rd sinus beat is conducted in a normal manner. 4th sinus beat is blocked in the AV node due to concealed conduction from previous extra systole resulting in a pause.

**Figure 3:**
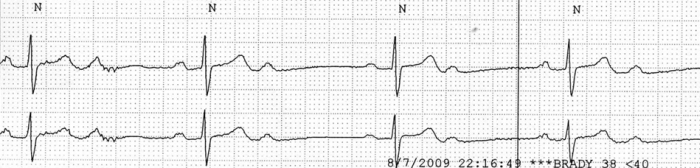
2:1 AV block on telemetry.

**Figure 4:**
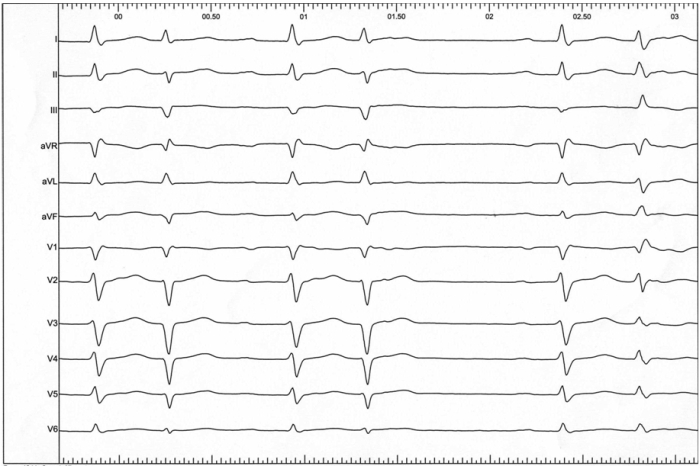
12 lead EKG at the time of electrophysiology study suggesting junctional bigeminy and compensatory pauses.

**Figure 5:**
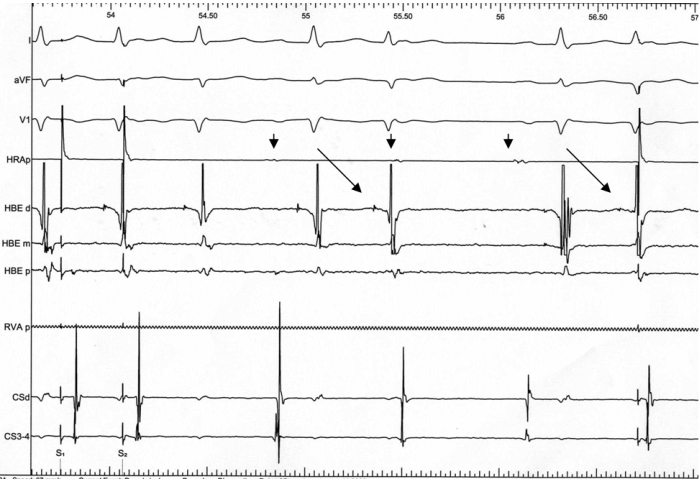
His Bundle electrograms between drive trains suggest His extra systoles (slanted arrows). Sinus atrial beats after his depolarization are blocked due to functional AV block. Note the HV interval is same for sinus depolarization and premature junctional depolarization.  Sinus beats (downward arrows) are uninterrupted by extra systoles.

A 56 year old male presented to our emergency room for recurrent episodes of presyncope. His past medical history was significant for hypertension and dyslipidemia. Physical examination, which included vital signs and orthostatic blood pressure measurements, were within normal limits on presentation. EKG suggested normal sinus rhythm at 75 bpm with first degree AV block and left anterior fascicular block (LAFB). He had frequent junctional premature complexes that would occasionally conduct with RBBB morphology due to rate related aberrancy (Ashman's phenomenon) (Figure1). A ladder diagram constructed to explain the beats between pauses illustrates concealed conduction from His extra systoles (Figure 2). The patient was not taking any AV nodal blocking agents. He developed 2:1 AV block with symptoms on inpatient telemetry (Figure 3). Echocardiogram suggested normal LV function with no evidence of structural heart disease. He had good exercise capacity and was able to exercise 9 minutes per Bruce protocol with no evidence of AV block. Myocardial perfusion imaging was negative for ischemia. He underwent an electrophysiology study which suggested frequent His extra systole with manifest functional AV block on surface EKG (Figure 4, 5). The patient's HV interval was 56 msec and was able to maintain 1:1 AV conduction until 150 bpm. 2:1 AV block in our patient can be explained by concealed retrograde conduction of His extra systole with functional AV block along with anterograde block in the His- Purkinje system. Our patient was treated with propafenone. His symptoms improved and outpatient event monitoring suggested rare instances of junctional premature complexes without AV block.

## Discussion

2:1 AV block presents a unique challenge to clinicians as the level of block can be difficult for localization [1]. It is important to determine the level of block as it can suggest prognosis and the need for a permanent pacemaker. More often an invasive electrophysiology study may be needed to determine the level of block. There are a few clues on the surface EKG which can be suggestive of infra or intra hisian block, such as a wide QRS complex and a PR interval <160msec. However 2:1 block can be functional due to premature atrial, junctional and ventricular depolarization with concealed conduction into the AV node or His bundle [2]. His bundle extra systoles can be completely concealed with no representation on surface EKG [3].

In patients with concealed AV block, pacemaker implantation doesn't improve prognosis but can worsen symptoms. Pacemakers normally consider a ventricular event without a preceding atrial event as premature depolarization. Most devices have inbuilt algorithms to extend PVARP (post ventricular atrial refractory period) to prevent pacemaker mediated tachycardia. Functional atrial unsensing and pacing in the atrial refractory period can commonly lead to repetitive non reentrant VA synchrony (RNVAS) [4]. This phenomenon can lead to unfavorable hemodynamics and syncope in such patients.

His bundle extra systoles can be due to abnormal automaticity or triggered activity. Most of the premature depolarizations can be successfully treated with anti arrhythmic drugs. However, with the higher prevalence of intra hisian block in patients with His extra systoles, a combination of anti arrhythmic medications or catheter ablation with pacemaker implantation is commonly required. Often on surface EKG frequent junctional premature complexes with occasional 2:1 AV block can be a clue to pseudo AV block.
